# Effectiveness of Live Poultry Market Interventions on Human Infection with Avian Influenza A(H7N9) Virus, China 

**DOI:** 10.3201/eid2605.190390

**Published:** 2020-05

**Authors:** Wei Wang, Jean Artois, Xiling Wang, Adam J. Kucharski, Yao Pei, Xin Tong, Victor Virlogeux, Peng Wu, Benjamin J. Cowling, Marius Gilbert, Hongjie Yu

**Affiliations:** Fudan University School of Public Health, Shanghai, China (W. Wang, X. Wang, Y. Pei, X. Tong, H. Yu);; Université Libre de Bruxelles, Brussels, Belgium (J. Artois, M. Gilbert);; London School of Hygiene and Tropical Medicine, London, UK (A.J. Kucharski);; École Normale Supérieure de Lyon, Lyon, France (V. Virlogeux);; Cancer Research Center of Lyon, Lyon (V. Virlogeux);; School of Public Health, University of Hong Kong, Hong Kong, China (V. Virlogeux, P. Wu, B.J. Cowling);; Fonds National de la Recherche Scientifique, Brussels (M. Gilbert)

**Keywords:** influenza in humans, influenza A(H7N9), live poultry markets, effectiveness assessment, viruses, China, influenza, avian influenza, zoonoses

## Abstract

Various interventions for live poultry markets (LPMs) have emerged to control outbreaks of avian influenza A(H7N9) virus in mainland China since March 2013. We assessed the effectiveness of various LPM interventions in reducing transmission of H7N9 virus across 5 annual waves during 2013–2018, especially in the final wave. With the exception of waves 1 and 4, various LPM interventions reduced daily incidence rates significantly across waves. Four LPM interventions led to a mean reduction of 34%–98% in the daily number of infections in wave 5. Of these, permanent closure provided the most effective reduction in human infection with H7N9 virus, followed by long-period, short-period, and recursive closures in wave 5. The effectiveness of various LPM interventions changed with the type of intervention across epidemics. Permanent LPM closure should be considered to maintain sufficient effectiveness of interventions and prevent the recurrence of H7N9 epidemics.

Human infections with avian influenza A(H7N9) virus were laboratory confirmed in China in the spring of 2013 (*1*). Since then, 1,567 human cases and 615 fatal cases have been officially reported in 5 epidemic waves (February–September 2013, October 2013–September 2014, October 2014–September 2015, October 2015–September 2016, and October 2016–September 2017) as of March 2, 2018 (*2*). Compared with the previous 4 epidemic waves, the 2016–17 fifth wave raised global concerns because of several characteristics. First, a surge in laboratory-confirmed cases of H7N9 virus infection was observed in wave 5, along with some clusters of limited human-to-human transmission (*3*,*4*). Second, a highly pathogenic avian influenza H7N9 virus infection was confirmed in Guangdong Province and has caused further human infections in 3 provinces (*5*,*6*). The genetic divergence of H7N9 virus, its geographic spread (*7*), and a much longer epidemic duration raised concerns about an enhanced potential pandemic threat in 2016–17.

Live poultry markets (LPMs) are a major source of human infections with H7N9 virus; the maintenance, amplification, and dissemination of H7N9 viruses have occurred in LPMs (*8*,*9*). Most human patients were exposed to H7N9 viruses through direct exposure to infected poultry or indirect exposure in contaminated environments, which increased the risk of H7N9 infections (*9*). Closure of LPMs is thus considered to play a key role in reducing the risk of animal-to-human transmission of H7N9. Different levels of LPM interventions were implemented in different geographic areas during 2013–2018. Permanent and temporary LPM closures were the main measures used to reduce the exposures of human population to H7N9 virus and reduce transmission (*10*,*11*). In some counties, alternative practices to complete bans of LPMs have also been put in place, such as bans on overnight poultry storage combined with regular cleaning and disinfection or market rest days (*12*).

So far, the effectiveness of LPMs interventions in controlling H7N9 epidemics has been discussed in several studies. In comparison with the previous 4 epidemic waves, a quantitative effectiveness assessment of LPM closure on the fifth H7N9 epidemic wave has not yet been conducted. Moreover, previous studies investigated the effect of the occurrence of LPM closure on controlling the H7N9 epidemic only by directly comparing the detection and isolation rates of H7N9 virus in the environment (*13*,*14*), investigating the number of H7N9 cases (*10*,*15*), or evaluating the posterior estimates of H7N9 incidence using transmission models before and after LPM closure (*16*–*18*). Although such modeling studies have quantified the effectiveness of LPM closure, inaccurate estimates of the effectiveness may have arisen because they did not account for the full characteristics of the LPM interventions (e.g., the type, start date, and duration of the interventions) and the underlying natural transmission dynamics of H7N9. In particular, neglecting the natural transmission dynamics of H7N9 may have led to underestimates or overestimates of the effectiveness of LPM closure if the interventions were implemented before or after the epidemic peak. Given the limitations of previous studies and variations in the implementation of LPM interventions in different geographic areas, there is a need to consider the potential effects of the characteristics of various interventions on the control of H7N9 epidemics.

Our study aimed to assess the differences in the effectiveness of various LPM interventions across 5 epidemic waves, especially during the 2016–17 epidemic wave. Specifically, we compared 4 LPM interventions: permanent, long-period, short-period, and recursive closures. We compared the daily incidence rates of H7N9 for different types and closing levels of LPM closure across 5 epidemic waves and quantified the effect of 4 LPM interventions on H7N9 transmission in the 2016–17 epidemic wave.

## Materials and Methods

### Data Sources

**Figure 1:**
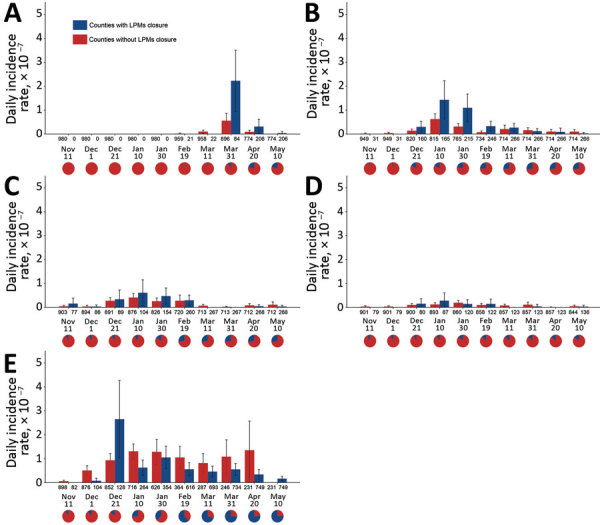
Mean daily incidence rates in counties with and without live poultry market closures across waves of influenza A(H7N9) infections, China, 2013–2017. A) Wave 1; B) wave 2; C) wave 3; D) wave 4; E) wave 5. Wave-specific calculations include only counties with >1 human case in that wave. Error bars indicate 95% CIs. Numbers below the axis represent the number of counties with and without LPM live poultry market closure at corresponding intervals; pie charts represents the proportion of counties with live poultry market closures at corresponding intervals. The timespan in the last interval was equal to the period from the end of the former interval to the date of infection of the last case in each wave.

We compiled a database recording the characteristics (e.g., the type, start date, and end date) of LPM closure (Appendix). We initially identified 32 types of LPM closure in cities with >1 H7N9 case (Appendix Table 1, Figure 1) and classified them based on the duration of LPM closure and the proportion of closing days. The duration of LPM closure refers to the total number of closing days; the proportion of closing days is equal to the duration of LPM closure divided by the duration of each epidemic wave. Given variations in duration, start dates, and end dates of the 5 H7N9 epidemic waves, it was not reasonable to use similar start and end dates for all epidemic waves to estimate daily incidence rates (DIRs). To give more comparable estimates of DIRs, we set the duration of each epidemic as the period separating the 5th from the 95th percentiles of the days of onset of illness in each wave. First, taking the duration of closure into consideration, we classified LPM closure measures into 4 categories: permanent closure, whereby LPMs were permanently closed within the epidemic wave or for the entire epidemic wave duration; long-period closure (>14 days within the epidemic wave [*10*,*17*]); short-period closure (<14 days within the epidemic wave); and recursive closure, whereby LPMs were closed for 1 or 2 day with a repetition of the closing over time (the closing might be implemented weekly, biweekly, or monthly). Second, we classified LPM closures according to the proportion of days of closing out of the total epidemic wave duration, using a quantile classification method (i.e., <25%, 25%–75%, and >75% of epidemic wave duration) because of abnormal distributions of the proportions of closing days in waves 1–5 (Appendix Figure 2). We collected the onset date and information on residence for all laboratory-confirmed H7N9 human cases during March 2013–September 2017 from the World Health Organization (https://www.who.int/csr/don/17-january-2017-ah7n9-china), Monthly Risk Assessment Summary reports (https://www.who.int/influenza/human_animal_interface/avian_influenza/archive), websites of the national and provincial Health and Family Planning Commission of China (http://www.nhc.gov.cn), FluTrackers (http://www.flutrackers.com), HealthMap (https://healthmap.com.au), and avian influenza reports from the Centre of Health Protection of Hong Kong (https://www.chp.gov.hk/tc/index.html).

### Statistical Analyses

#### Assessment of Type of LPM Closure on H7N9 DIR

We first assessed the effect of 4 types of LPM closures (recursive, short-period, long-period, and permanent closures) on H7N9 DIRs. We calculated DIR estimates only for counties where >1 H7N9 case was reported in 2013–2017 (Appendix). In addition to looking at the type of the intervention, we also explored the influence of the closing levels of LPM closure (<25%, 25%–75%, and >75% of epidemic wave duration) on DIRs. We used a generalized linear mixed effect model (GLMM) followed by a multiple comparison procedure (Tukey test) to compare DIRs by contrasting counties with no measures to counties with different types and closing levels of LPM closure before and after these measures were taken.

#### Assessment of LPM Interventions on Risk for Animal-to-Human and Human-to-Human Transmission in the 2016–17 Epidemic Wave

To further assess the effect of the type of LPM closure on reduction in H7N9 transmission risk in each site, we constructed an H7N9 transmission model similar to that developed by Yu et al. (*16*) and Virlogeux et al. (*18*) using data from the 2016–17 epidemic wave (Appendix). We included 17 sites (60 districts/counties) with >5 urban and semiurban cases in wave 5 (Appendix Figure 3). We compared the reduction in the number of animal-to-human infections before and after closure among 4 LPM interventions using Welch’s analysis of variance and multiple comparison (Tamhane’s T2 test).

The H7N9 epidemics in 2013–2017 followed a seasonal pattern, with peaks in the winter months and sporadic cases in the summer months. Thus, we considered the reductions in number of infections, together with LPM interventions, to be correlated with the seasonal pattern of the H7N9 epidemics. We incorporated absolute humidity, the most dominant contributor to the H7N9 epidemic, into transmission models to modulate the seasonal pattern of H7N9 epidemic in a sensitivity analysis (Appendix) (*19*,*20*). We assumed the transmissibility of H7N9 virus to be higher at lower absolute humidity in accordance with previous studies (*19*,*20*) and an observed pattern of H7N9 epidemic in the 17 study sites (Appendix Figure 4). In addition, we separated the effect of LPM closure from the natural transmission dynamics of H7N9 viruses by comparing the differences in the reductions in the number of infections between 2 sites (1 with and 1 without LPM closure) where a similar season pattern of human H7N9 infections had been observed. We created hypothetical start and end dates of LPM closures in sites without such closures and assumed them to be consistent with those in sites with closures. We used the Mann-Whitney U test to compare the differences of the reductions in the number of infections between the 2 sites.

## Results

**Figure 2:**
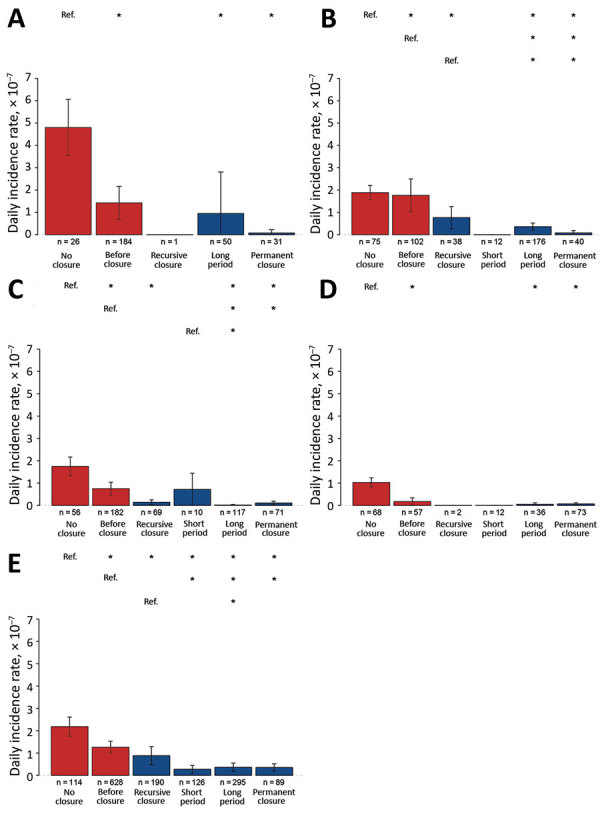
Estimated daily incidence rates in counties with various levels of live poultry market closures across waves of influenza A(H7N9) infections, by duration of closure, China, 2013–2017. A) Wave 1; B) wave 2; C) wave 3; D) wave 4; E) wave 5. Error bars indicate 95% CIs. Asterisks (*) above bars indicate statistically significant (p<0.05) differences between daily incidence rates and reference category (Ref.) rates. Duration categories: no closure during epidemic wave; permanent closure, permanently closed within the epidemic wave or for the entire epidemic wave duration; long-period closure (>14 days within the epidemic wave [*10**,**17*]); short-period closure (<14 days within the epidemic wave); and recursive closure, whereby LPMs were closed for 1 or 2 day with a repetition of the closing over time (the closing might be implemented weekly, biweekly, or monthly).

**Figure 3:**
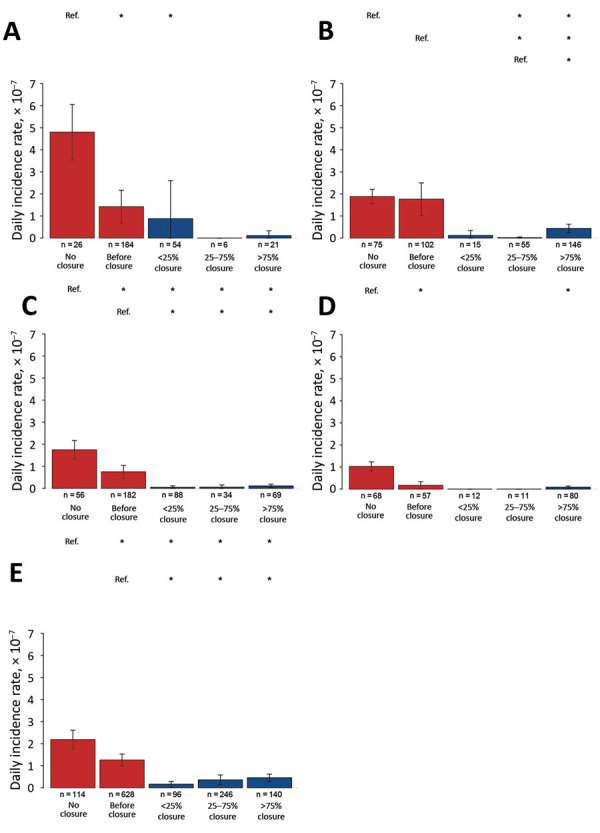
Estimated daily incidence rates in counties with various levels of live poultry market (LPM) closures across waves of influenza A(H7N9) infections, by proportion of closure days during epidemic wave, China, 2013–2017. A) Wave 1; B) wave 2; C) wave 3; D) wave 4; E) wave 5. Error bars indicate 95% CIs. Asterisks (*) above bars indicate statistically significant (p<0.05) differences between daily incidence rates and reference category (Ref.) rates. Proportion categories: no closure; before closure, incidence rate before market was closed; <25%, closed <25% of the days of the wave duration; 25%–75%, closed 25%–75% of the days of the wave duration; >75%, closed >75% of the days of the wave duration.

The comparison over time of DIRs between counties with and without LPM closures (Figure 1) showed that counties with measures had higher DIRs than counties free of closures during 2013–2017. In wave 5, DIRs decreased over time in counties with closures, whereas DIRs for counties without measures remained fairly high. Comparisons of DIRs for counties with different types (Figure 2) and levels (Figure 3) of LPM closure showed that, with the exception of wave 1 and wave 4, showed that DIRs were significantly lower in counties after closure than before (p<0.001) (Appendix Table 2). The DIRs in counties after LPM closure were also significantly lower than those estimated for counties without closures (p<0.001) (Appendix Table 2). We observed no statistically significant difference between counties with recursive, short-period, long-period, or permanent closures except for counties with recursive, long-period, and permanent closures in wave 2; for counties with short-period and long-period closures in wave 3; and for counties with recursive and long-period closures in wave 5. No DIRs were significantly different among counties with different levels of closing days, but the difference was significant in 25%–75% versus >75% of epidemic wave duration in wave 2.

To further quantify the effectiveness of LPM intervention in each site in wave 5, we compared the reduction in number of daily infections before and after closure among counties with 4 LPM interventions. A total of 142 laboratory-confirmed cases were located in 17 sites in wave 5 (Table 1), which is much higher than the total number of H7N9 cases in these sites in waves 1–4 (n = 116). A compilation of the onset dates of illness for these cases (Appendix Figure 5) shows that, with the exception of 4 study sites where human H7N9 epidemics ended before closing LPMs (study sites 8, 10–11, and 14), there was an observable drop in the number of H7N9 cases after LPM intervention in each site. After LPM closure, Gusu District in Suzhou, with permanent closure, had a higher reduction (97.0%, 95% CI 94.0%–100.0%) than other sites. The mean posterior estimates of the reductions ranged from 48% to 98% in sites with long-period closure. Guangzhou implemented recursive measures at the beginning of the epidemic but had a much lower reduction (34.0%, 95% CI 15.0%–70.0%). Compared with Guangzhou, which had short-period closures in the second intervention (73.0%, 95% CI 53.0%–77.0%), Foshan (96%) and Fuzhou (95%) showed larger relative reductions in the daily number of infections. Overall, the mean reduction in daily number of infections increased successively among sites with recursive, short-period, long-period, and permanent closures (p<0.001) (Appendix Table 3).

**Table 1:** Characteristics of study sites in the 2016–17 epidemic wave of influenza A(H7N9), China.

Province	City	District/county, n = 60	Site no., n = 17	No. cases		Type of LPM closure	LPM closures	
				Urban	Semiurban		Start date	End date
Jiangsu	Suzhou	Gusu District	1	19	1	Permanent	2016 Dec 31	Unreported
		Huqiu District, Wujiang District, Wuzhong District, Xiangcheng District	2	12	6	Long-period	2016 Dec 27	Unreported
		Kunshan City	3	5	1	Long-period	2016 Dec 19	Unreported
	Wuxi	Xishan District, Binhu District, Huishan District, Liangxi District (Chongan District, Nanchang District, Beitang District), Xinwu District, Jiangyin City	4	9	8	Long-period	2016 Dec 29	2017 Apr 27
	Changzhou	Zhonglou District, Tianning District, Wujin District, Xinbei District, Jintan City, Liyang City	5	8	4	Long-period	2016 Dec 30	2017 Apr 30
	Nantong	Chongchuan District	6	5	0	Long-period	2017 Feb 25	Unreported
Guangdong	Guangzhou	Haizhu District, Tianhe District, Panyu District, Baiyun District	7	5	3	Recursive	2017 Jan 1	2017 Feb 15
			7			Short-period	2017 Feb 16	2017 Feb 28
	Foshan	Nanhai District, Shunde District	8	1	4	Short-period	2017 Jan 16	2017 Jan 25
Zhejiang	Ningbo	Yuyao City, Cixi City, Fenghua City, Ninghai County	9	1	8	Long-period	2017 Feb 11	Unreported
	Hangzhou	Yuhang District, Xiaoshan District, Linan City, Fuyang City, Chunan County	10	2	5	Long-period	2017 Feb 11	Unreported
	Wenzhou	Dongtou District, Yueqing City, Ruian County, Cangnan County	11	1	4	Long-period	2017 Feb 11	Unreported
	Lishui	Suichang County, Jingning County, Jinyun County, Qingyuan County	12	0	5	Long-period	2017 Feb 11	Unreported
Hunan	Xiangtan	Yuetang District, Yuhu District, Xiangtan County	13	3	2	Long-period	2017 Jan 24	Unreported
Anhui	Suzhou City	Yongqiao District	14	5	0	Long-period	2017 Feb 15	2017 Apr 30
Fujian	Fuzhou	Jinan District, Gulou District, Taijiang District	15	5	0	Short-period	2017 Feb 7	2017 Feb 17
Sichuan	Aba	Jinchuan County, Ruoergai County, Xiaojin County	16	0	5	Recursive	2017 May 10	Unreported
Shanghai	Shanghai	Chongming District, Fengxian District, Jiading District, Jingan District, Jinshan District	17	2	3	Long-period	2017 Jan 28	2017 Apr 30

*Unreported indicates that the end of the LPM closure was not observed before May 31, 2017. LPM, live poultry market.

When we examined potential for human-to-human transmission, we found that the estimated effective reproduction number was 0.147 (95% CI 0.034–0.285) (Table 2; Appendix Figure 6). The slightly higher daily number of infections estimated by the model incorporating animal-to-human and human-to-human transmission (Appendix Figure 5) also suggests the potential for human-to-human transmission when compared with those estimates in an animal-to-human transmission model (Appendix Figure 7). Sensitivity analyses examined the influence of mean serial interval and of the proportion of unreported cases on the effective reproduction number. A decrease in the effective reproduction number was observed when the mean serial interval increased (Appendix Table 4). After accounting for the seasonality of H7N9 affected by absolute humidity, estimates of the reduction in number of daily infections changed slightly in some sites (Appendix Table 5), which should not be surprising, because the season pattern of H7N9 epidemics may well vary from one site to another (Appendix Figure 4). After we adjusted for the potential effect of the natural transmission dynamics of H7N9 virus, the net effect of LPM closure varied in study sites with long-period (range 0.5%–52.0%) and permanent (45.0%, 95% CI 32.0%–88.0%) closures in wave 5 (Table 3). In all study sites except 1, the differences in reductions in number of infections among sites with and without closures were statistically significant (p<0.001).

**Table 2:** Parameter estimation of infection rates before and after live poultry market closures in the 2016–17 influenza A(H7N9) epidemic wave, China.

Site no.	Type of closure	Expected daily no. infections (95% CI)		Reduction in no. infections after closure, % (95% CI)	Reproduction number (95% CI)
		Before closure	After closure		
1	Permanent	0.230 (0.121–0.372)	0.006 (0.000–0.023)	97.0 (94.0–100.0)	0.147 (0.034–0.285)
2	Long	0.340 (0.171–0.540)	0.034 (0.009–0.073)	90.0 (87.0–95.0)	
3	Long	0.120 (0.037–0.248)	0.010 (0.000–0.033)	92.0 (87.0–100.0)	
4	Long	0.390 (0.183–0.648)	0.018 (0.000–0.064)	95.0 (90.0–100.0)	
5	Long	0.460 (0.231–0.763)	0.008 (0.000–0.033)	98.0 (96.0–100.0)	
6	Long	0.040 (0.014–0.089)	0.022 (0.003–0.058)	48.0 (35.0–81.0)	
7	Recursive	0.162 (0.037–0.229)	0.107 (0.012–0.301)	34.0 (15.0–70.0)	
7	Short	0.107 (0.012–0.301)	0.029 (0.005–0.068)	73.0 (53.0–77.0)	
8	Short	0.190 (0.062–0.379)	0.008 (0.000–0.028)	96.0 (93.0–99.0)	
9	Long	0.120 (0.050–0.220)	0.019 (0.002–0.055)	84.0 (75.0–96.0)	
10	Long	0.090 (0.032–0.176)	0.001 (0.000–0.035)	89.0 (80.0–99.0)	
11	Long	0.110 (0.037–0.229)	0.009 (0.000–0.035)	92.0 (84.0–99.0)	
12	Long	0.090 (0.029–0.193)	0.020 (0.002–0.054)	78.0 (72.0–92.0)	
13	Long	0.220 (0.048–0.518)	0.031 (0.008–0.069)	86.0 (83.0–87.0)	
14	Long	0.190 (0.066–0.374)	0.016 (0.000–0.052)	92.0 (86.0–100.0)	
15	Short	0.350 (0.117–0.728)	0.019 (0.002–0.055)	95.0 (92.0–98.0)	
16	Recursive	0.210 (0.068–0.400)	0.060 (0.002–0.211)	71.0 (47.0–97.0)	
17	Long	0.140 (0.044–0.295)	0.022 (0.003–0.062)	84.0 (79.0–94.0)	


**Table 3:** Estimates of the net effect of LPM closures by comparing the reductions in the number of influenza A(H7N9) infections between study sites with and without closures, adjusting for similar season pattern of absolute humidity, China*

Study sites with LPM interventions					Reduction in no. infections, % (95% CI)			
	Reference sites without LPM interventions				Study sites with LPM interventions, R_1_	Reference sites without LPM interventions, R_2_	Difference in reduction, R_1_ – R_2_	p value
	Site no.	Province	Cities					
Site 1	1	Jiangsu	Huaian (Huaian District, Qingpu District), Nantong (Haimen City, Rugao City), Xuzhou (Suining County), Yancheng (Dongtai City), Yangzhou (Hanjiang District)		97.0 (93.0–100.0)	52.0 (12.0–61.0)	45.0 (32.0–88.0)	<0.001
Site 2	1				90.0 (88.0–94.0)	57.0 (16.0–67.0)	33.0 (21.0–78.0)	<0.001
Site 3	1				90.0 (84.0–99.0)	70.0 (42.0–76.0)	20.0 (8.0–57.0)	<0.001
Site 4	1				94.0 (89.0–99.0)	49.0 (10.0–48.0)	44.0 (30.0–89.0)	<0.001
Site 5	1				98.0 (96.0–100.0)	48.0 (20.0–58.0)	51.0 (38.0–80.0)	<0.001
Site 6	1				50.0 (32.0–74.0)	32.0 (21.0–50.0)	18.0 (10.0–23.0)	<0.001
Site 14	1				93.0 (86.0–100.0)	40.0 (27.0–63.0)	52.0 (36.0–59.0)	<0.001
Site 17	1				84.0 (78.0–94.0)	46.0 (39.0–61.0)	38.0 (33.0–39.0)	<0.001
Site 13	2	Hunan	Chenzhou (Beihu District, Yongxing County), Hengyang (Hengdong County, Shigu District, Zhuhui District), Loudi (Shuangfeng District), Shaoyang (Shaodong County, Xinning County, Xinshao County), Zhangjiajie (Yongding District)		86.0 (81.0–87.0)	85.0 (84.0–86.0)	0.5 (−3.0 to 1.0)	0.098

*Similar seasonal patterns of absolute humidity had been observed among study sites with and without closures (e.g., study site 1–6, site 14, site 17, and reference site 1). LPM, live poultry market.

## Discussion

LPM closing measures have often been implemented reactively, after the occurrence of human H7N9 cases in a given county (Appendix Figure 8); it is thus not surprising to find generally high DIRs in counties that undertook such measures (Figure 1). However, what matters most is what happened to the DIR and mean daily number of illnesses after these closing measures were taken. Both DIRs and mean daily number of illness onsets decreased in counties or sites following LPM interventions, but the effect varied depending on the type of intervention and epidemic wave.

In general, permanent, long-period, and short-period closures provided comparable estimates in terms of DIR reduction. However, the association between the type and closing levels of LPM measures and DIRs showed different results across waves. For example, the difference in DIRs in counties with different levels of closing days was observed only in wave 2. During this wave, long-period and permanent closures represented the large majority of the measures (82.4% of the closing measures). During wave 5, short-period and recursive closures became available to authorities as potential measures and were implemented more abundantly, especially in cities with few H7N9 cases; thus, long-period and permanent closures represented only 55% of the total closing measures.

For wave 5, we also evaluated the effectiveness of different types of LPM interventions in controlling H7N9 epidemics in several key sites. Overall, the effectiveness of LPM closure varied with the type of the interventions in these sites during 2016–17. Permanent closure was more effective than long-period closure, short-period closure, and recursive closure. The relatively lower effectiveness of short-period closure was observed in wave 5, but the point estimates of the reduction in daily number of infections inferred from the transmission model were consistent with the effectiveness assessment of a 14-day LPM closure (range 53.0%–89.0%) (*17*). Accompanying the effectiveness assessment of consecutive LPM closure, Yuan et al. (*21*) quantified the effectiveness of periodic LPM closure together with daily cleaning and disinfection (range −47.0% to 34.0%), which was consistent with our minimum point estimates of the effectiveness of recursive closure.

The decline in the number of human infections with H7N9 virus varied among study sites. In addition to being a factor of the type of the intervention, the variations in these declines may have been influenced by the underlying natural transmission dynamics of H7N9. After adjusting for absolute humidity, the most dominant environmental driver for influenza seasonality, the reduction in number of infections did not change significantly in any one of the study sites. Therefore, overall estimates of the effect of LPM closure is unlikely to be confounded by those climatic factors. However, we cannot exclude the possibility that the effectiveness of LPM closure may be delayed because of climatic factors at a specific time, as low temperature and higher humidity always drive the spread of H7N9 virus. In addition, we cannot definitively exclude other unknown seasonal confounders, such as the seasonality of poultry movement. Available evidence supports the seasonal effects of poultry movement on human infection with H5N1 virus around Chinese New Year (*22*). Although we found no quantitative evidence that seasonal variation in poultry trade played a role in human infection with H7N9 virus, the fact that the high-risk season of the H7N9 epidemic was consistent with the peak time of poultry trade around Chinese New Year is notable.

Limited human-to-human transmissibility of H7N9 virus was previously observed during waves 1–4 (*3*). Our low estimates of reproduction number in wave 5 were consistent with previous descriptive analysis of possible clusters of human infection with H7N9 virus (*3*,*23*), confirming that human-to-human transmissibility of H7N9 virus remained unsustainable.

Other factors, such as societal economic costs and residents’ behavior toward banning live poultry trade, may affect the effectiveness of LPM closure (*24*) and lead to a displacement effect. LPM closure has threatened the wholesale and retail market chain (*25*); local authorities in epidemic areas even tried to control the spread of H7N9 virus by banning live poultry trade. Consequences of such interventions included loss of consumer confidence, decreases in prices of poultry products, and loss of market shares. In an attempt to reduce adverse effects in economic, less disruptive interventions were introduced, such as rest days, banning live poultry overnight, or periodic cleaning and disinfection (*26*,*27*). These LPM interventions proved to be less effective (*28*).

Besides LPM interventions, several key measures (e.g., culling known infected poultry and direct contacts, vaccinating poultry, or improving biosecurity for poultry-handling practices) have been taken to control zoonotic infection with H7N9 viruses (*29*). These measures are always applied in parallel and have gradually changed human behaviors related to the management, transportation, and trade of poultry. Specifically, traditional poultry handling and trade practices have been replaced by central slaughtering and frozen poultry products in major cities in China, which may have substantially reduced the risk of human exposure to infected poultry. The government of China implanted vaccination of poultry against H7N9 virus to control the 2017–18 epidemic wave after the surge in the reported number of cases in wave 5. The introduction of this H5/H7 bivalent inactivated vaccine substantially reduced the number of cases in the 2017–18 epidemic wave (*30*), although its effectiveness needs to be further assessed quantitatively.

This study has several limitations. First, the timing of the implementation of LPM closures in relation to the progress of the H7N9 epidemic was not considered in the effectiveness assessment of LPM closures, which would lead to an overestimation of the effects of LPM closure if LPM interventions were implemented after the epidemic peak. The incidence reduction might also not be comparable in cities with LPM interventions implemented before reaching the epidemic peak with those implemented near the end of the epidemic. Second, our findings focus only on human cases occurring in urban and semiurban areas in China in wave 5, ignoring H7N9 cases in rural areas, where LPMs are rarely located. More rural cases were reported in wave 5 than in previous epidemic waves, and exposure to poultry in farms and backyards were the main sources of these rural human cases (*9*,*31*). Therefore, LPM closure might be less effective in controlling H7N9 epidemics in these rural areas, and other effective interventions (e.g., vaccination of poultry) need to be further explored. Third, because of the ecologic nature of our study, some anthropogenic factors may have acted as potential confounders that can bias our findings, such as the number of LPM visitors, frequency of LPM visits, improvements in biosecurity for poultry-handling practices, or which live bird species were found in LPMs. These factors and LPM interventions have always existed in parallel, so we cannot rule out the possibility that differences in the reduction in the daily number of infections among different sites may be partially explained by these anthropogenic factors, especially in sites with recursive or short-period closures. To more precisely differentiate the effectiveness of each type of LPM interventions, future studies could incorporate additional datasets to try to separate the effects of LPM closure from the natural transmission dynamics of H7N9 virus and other anthropogenic factors. Furthermore, the estimate of the reproduction number in this study relies on the assumption that this parameter is constant among locations. Although the estimate of this parameter did not involve the geographic locations of these cases and the likelihood that these human cases might have been in contact, the estimate was consistent with previous epidemiologic studies (*3*,*23*).

A number of research questions need to be further clarified in future studies. Rhe optimal time and duration to implement LPM closure to balance the economic loss and transmission risk reduction needs further investigation, combined with a time-varying force of infection. Moreover, it could be possible to estimate key epidemiologic parameters (e.g., animal-to-human transmissibility and reproduction number) by considering the spatial–temporal dynamics of H7N9 epidemics in poultry and related environments, potential market functioning effects, and the frequency of human exposure to H7N9 virus to explain the differences in effectiveness.

In conclusion, the characteristics of LPM interventions can potentially affect their effectiveness. Although possibly more challenging from an operational point of view, permanent and long-period closures were found to be more effective in reducing human H7N9 cases during waves 1–5. In the long term, structural changes in the poultry value chain linked to permanent LPM closure may be required to maintain sufficient effectiveness of interventions and prevent the occurrence of H7N9 epidemics.

Appendix Additional information on the study of the effectiveness of live poultry market closures on human H7N9 infection, China, 2013–2017.

## References

[1] Liu D, Shi W, Shi Y, Wang D, Xiao H, Li W, et al. Origin and diversity of novel avian influenza A H7N9 viruses causing human infection: phylogenetic, structural, and coalescent analyses. Lancet. 2013;381:1926–32. 10.1016/S0140-6736(13)60938-1 23643111

[2] World Health Organization. Human infection with avian influenza A(H7N9) virus—China. 2019 [cited 2019 Nov 23]. https://www.who.int/csr/don/13-september-2017-ah7n9-china

[3] Zhou L, Chen E, Bao C, Xiang N, Wu J, Wu S, et al. Clusters of human infection and human-to-human transmission of avian influenza A(H7N9) virus, 2013–2017. Emerg Infect Dis. 2018;24:397–400. 10.3201/eid2402.171565 29165238 PMC5782887

[4] Bertran K, Balzli C, Kwon YK, Tumpey TM, Clark A, Swayne DE. Airborne transmission of highly pathogenic influenza virus during processing of infected poultry. Emerg Infect Dis. 2017;23:1806–14. 10.3201/eid2311.170672 29047426 PMC5652435

[5] Centers for Disease Control and Prevention. Mutations on H7N9 virus have been identified in H7N9 patients in China. 2017 [cited 2018 Apr 20]. http://www.chinacdc.cn/jkzt/crb/zl/rgrgzbxqlgg/rgrqlgyp/201702/t20170219_138185.html

[6] Qi W, Jia W, Liu D, Li J, Bi Y, Xie S, et al. Emergence and adaptation of a novel highly pathogenic H7N9 influenza virus in birds and humans from a 2013 human-infecting low-pathogenic ancestor. J Virol. 2018;92:e00921-17. 10.1128/JVI.00921-17 29070694 PMC5752946

[7] Artois J, Jiang H, Wang X, Qin Y, Pearcy M, Lai S, et al. Changing geographic patterns and risk factors for avian influenza A(H7N9) infections in humans, China. Emerg Infect Dis. 2018;24:87–94. 10.3201/eid2401.171393 29260681 PMC5749478

[8] Lam TT, Wang J, Shen Y, Zhou B, Duan L, Cheung CL, et al. The genesis and source of the H7N9 influenza viruses causing human infections in China. Nature. 2013;502:241–4. 10.1038/nature12515 23965623 PMC3801098

[9] Wang X, Jiang H, Wu P, Uyeki TM, Feng L, Lai S, et al. Epidemiology of avian influenza A H7N9 virus in human beings across five epidemics in mainland China, 2013-17: an epidemiological study of laboratory-confirmed case series. Lancet Infect Dis. 2017;17:822–32. 10.1016/S1473-3099(17)30323-7 28583578 PMC5988584

[10] He Y, Liu P, Tang S, Chen Y, Pei E, Zhao B, et al. Live poultry market closure and control of avian influenza A(H7N9), Shanghai, China. Emerg Infect Dis. 2014;20:1565–6. 10.3201/eid2009.131243 25148432 PMC4178391

[11] Friedrich MJ. Closing live poultry markets slowed avian flu in China. JAMA. 2013;310:2497. 10.1001/jama.2013.283885

[12] Liu H, Chen Z, Xiao X, Lu J, Di B, Li K, et al. [Effects of resting days on live poultry markets in controlling the avian influenza pollution]. Zhonghua Liu Xing Bing Xue Za Zhi. 2014;35:832–6. 25294077

[13] Kang M, He J, Song T, Rutherford S, Wu J, Lin J, et al. Environmental sampling for avian influenza A(H7N9) in live-poultry markets in Guangdong, China. PLoS One. 2015;10:e0126335. 10.1371/journal.pone.0126335 25933138 PMC4416787

[14] Yuan J, Lau EH, Li K, Leung YH, Yang Z, Xie C, et al. Effect of live poultry market closure on avian influenza A(H7N9) virus activity in Guangzhou, China, 2014. Emerg Infect Dis. 2015;21:1784–93. 10.3201/eid2110.150623 26402310 PMC4593444

[15] Cheng W, Wang X, Shen Y, Yu Z, Liu S, Cai J, et al. Comparison of the three waves of avian influenza A(H7N9) virus circulation since live poultry markets were permanently closed in the main urban areas in Zhejiang Province, July 2014-June 2017. Influenza Other Respir Viruses. 2018;12:259–66. 10.1111/irv.12532 29243376 PMC5820431

[16] Yu H, Cowling BJ, Liao Q, Fang VF, Zhou S, Wu P, et al. Effect of closure of live poultry markets on poultry-to-person transmission of avian influenza A H7N9 virus: an ecological study. Lancet 2014;383:541–8. 10.1016/S0140-6736(13)61904-2 24183056 PMC3946250

[17] Kucharski AJ, Mills HL, Donnelly CA, Riley S. Transmission potential of influenza A(H7N9) virus, China, 2013–2014. Emerg Infect Dis. 2015;21:852–5. 10.3201/eid2105.141137 25897624 PMC4412215

[18] Virlogeux V, Feng L, Tsang TK, Jiang H, Fang VJ, Qin Y, et al. Evaluation of animal-to-human and human-to-human transmission of influenza A (H7N9) virus in China, 2013-15. Sci Rep. 2018;8:552. 10.1038/s41598-017-17335-9 29323268 PMC5765021

[19] Li J, Rao Y, Sun Q, Wu X, Jin J, Bi Y, et al. Identification of climate factors related to human infection with avian influenza A H7N9 and H5N1 viruses in China. Sci Rep. 2015;5:18094. 10.1038/srep18094 26656876 PMC4676028

[20] Shaman J, Kohn M. Absolute humidity modulates influenza survival, transmission, and seasonality. Proc Natl Acad Sci U S A. 2009;106:3243–8. 10.1073/pnas.0806852106 19204283 PMC2651255

[21] Yuan J, Tang X, Yang Z, Wang M, Zheng B. Enhanced disinfection and regular closure of wet markets reduced the risk of avian influenza A virus transmission. Clin Infect Dis. 2014;58:1037–8. 10.1093/cid/cit951 24368621

[22] Soares Magalhães RJ, Zhou X, Jia B, Guo F, Pfeiffer DU, Martin V. Live poultry trade in Southern China provinces and HPAIV H5N1 infection in humans and poultry: the role of Chinese New Year festivities. PLoS One. 2012;7:e49712. 10.1371/journal.pone.0049712 23166751 PMC3500328

[23] Hu J, Zhu Y, Zhao B, Li J, Liu L, Gu K, et al. Limited human-to-human transmission of avian influenza A(H7N9) virus, Shanghai, China, March to April 2013. Euro Surveill. 2014;19:20838. 10.2807/1560-7917.ES2014.19.25.20838 24993556

[24] Liao Q, Yuan J, Lau EH, Chen GY, Yang ZC, Ma XW, et al. Live bird exposure among the general public, Guangzhou, China, May 2013. PLoS One. 2015;10:e0143582. 10.1371/journal.pone.0143582 26623646 PMC4666652

[25] Nicita A. Avian influenza and the poultry trade. 2008 [cited 2020 Feb 20]. https://elibrary.worldbank.org/doi/abs/10.1596/1813-9450-4551

[26] Lau EH, Leung YH, Zhang LJ, Cowling BJ, Mak SP, Guan Y, et al. Effect of interventions on influenza A (H9N2) isolation in Hong Kong’s live poultry markets, 1999-2005. Emerg Infect Dis. 2007;13:1340–7. 10.3201/eid1309.061549 18252105

[27] Wu P, Wang L, Cowling BJ, Yu J, Fang VJ, Li F, et al. Live poultry exposure and public response to influenza A(H7N9) in urban and rural China during two epidemic waves in 2013–2014. PLoS One. 2015;10:e0137831. 10.1371/journal.pone.0137831 26367002 PMC4569561

[28] Offeddu V, Cowling BJ, Malik Peiris JS. Interventions in live poultry markets for the control of avian influenza: a systematic review. One Health. 2016;2:55–64. 10.1016/j.onehlt.2016.03.002 27213177 PMC4871622

[29] Sims LD. Intervention strategies to reduce the risk of zoonotic infection with avian influenza viruses: scientific basis, challenges and knowledge gaps. Influenza Other Respir Viruses. 2013;7(Suppl 2):15–25. 10.1111/irv.12076 24034479 PMC5909387

[30] Shi J, Deng G, Ma S, Zeng X, Yin X, Li M, et al. Rapid evolution of H7N9 highly pathogenic viruses that emerged in China in 2017. Cell Host Microbe. 2018;24:558–68.e7. 30269969 10.1016/j.chom.2018.08.006 PMC6310233

[31] Chen E, Wang MH, He F, Sun R, Cheng W, Zee BCY, et al. An increasing trend of rural infections of human influenza A (H7N9) from 2013 to 2017: A retrospective analysis of patient exposure histories in Zhejiang province, China. PLoS One. 2018;13:e0193052. 10.1371/journal.pone.0193052 29447278 PMC5814046

