# Supplementary material for: Effectiveness of Live Poultry Market Interventions on Human Infection with Avian Influenza A(H7N9) Virus, China
Source: Emerg Infect Dis. 2020 May;26(5):891–901. doi: 10.3201/eid2605.190390 (PMC7181931; doi:10.3201/eid2605.190390)
Supplement: Appendix — Additional information on the study of the effectiveness of live poultry market closures on human H7N9 infection, China, 2013–2017. [file 19-0390-Techapp-s1.pdf]

Article DOI: https://doi.org/10.3201/eid2605.190390

Effectiveness of Live Poultry Market Interventions on Human Infection with Avian Influenza A(H7N9) Virus, China

Appendix

Live Poultry Market Closure Database

We obtained a database of live poultry market (LPM) closures at district or county levels through the official website of the Agricultural Bureaus and the Animal Husbandry and Veterinary Bureau at province and county levels; the municipal social media; and internet searches through the Baidu, Sougou, and Bing search engines by using the search terms “live poultry market closure,” “live poultry market,” “live poultry trade,” and “H7N9-positive,” with predefined locations corresponding to the prefectures with $\geq$1 H7N9 case over the 5 epidemics (Appendix Figure 1). Two independent investigators applied the same search procedure for cross-checking and comprehensiveness.

Assessment of Type of LPM Closure on H7N9 Daily Incidence Rate

We defined the H7N9 daily incidence rate (DIR) as the number of new cases during the timespan / (population × total number of days during the timespan). We defined the timespan differently according to the dates of the first LPM closure. In counties without LPM closure, the timespan was the duration of the epidemic wave (i.e., the 5th to the 95th percentiles of the days of onset of illness in each wave). In counties with LPM closure, we considered 2 timespans: the first was the period preceding the implementation of the first LPM closure and the second was the period after the implementation of the first measure until the 95th percentiles of the days of

onset of illness. We used the proportion of closing days after the first LPM closure to contrast different levels of closures in that period: low (<25% of closing days), intermediate (25–75% of closing days), and high (>75% of closing days).

The generalized linear mixed effect models (GLMMs) were formulated with a Poisson distribution taking the county level as random effects (2 observations of the same county, before and after the closure, may be considered separately in the models and represent a bias to the assumption of independence of observations). In addition, DIRs estimated from very short periods of time may have extreme variation because of the stochasticity of case reports. Therefore, the GLMM included DIR estimates only for durations >20% of the full epidemic wave duration. We excluded groups with <20 LPM closures from multiple comparison procedures considering GLMM convergence. We compared the performances of GLMMs with various types and closing levels using the Akaike information criterion.

Assessment of LPM Interventions on Risk of Animal-to-Human and Human-to-Human Transmission in 2016–2017 Epidemic

Study Site Selection

We initially included 271 districts/counties in 26 cities with ≥5 urban and semiurban human H7N9 cases in the 2016–2017 epidemic. Of these, we excluded 211 districts/counties for 1 of 3 reasons: permanent closure before the fifth wave, without H7N9 cases, or without LPMs closure (Appendix Figure 3). We aggregated the onset data in the remaining 60 districts/counties based on the same LPM closure measure within the same cities.

Transmission Model

In the transmission model, we assumed human cases to be generated by two processes: index cases infected from animal exposure and secondary cases generated by previous infections. Therefore, the expected number of human cases with onset of day t depends on the animal-to-

human transmission function $h_{A,i}(t)$ and 2 human-to-human transmission parameters: mean serial interval (the time between successive cases in a chain of transmission, S_p) and the effective reproduction number (R_e). A similar approach has been applied and validated in other modeling studies (*1*,*2*).

Animal-to-Human Transmission Model

In study sites, the mean incubation period of H7N9 infection was assumed to be 3.3 days and followed the same probability Weibull distribution F with scale μ and shape σ for all study sites (*3*).

For study sites with 1 LPM closure measure, the new animal-to-human infections in study site *i* followed a Poisson distribution with mean $\lambda_{pre,i} = p_i \cdot \pi_{pre,i}$ for $t \in [t_a, t_b)$, and $\lambda_{post,i} = p_i \cdot \pi_{post,i}$ for $t \in [t_b, t_c)$ where p_i was the ascertainment proportion associated with the confirmed cases. We thus defined the transmission function $h_{A,i}(t)$ to be the number of cases due to exposure to animals in study site *i*:

$$h_{A,i}(t) = \begin{cases} F(t) \cdot \lambda_{pre,i} & t \in [t_a, t_b) \\ F(t-t_b) \cdot \lambda_{post,i} + [1 - F(t-t_b)] \cdot \lambda_{pre,i} & t \in [t_b, t_c) \end{cases}$$

where $\lambda_{pre,i}$ and $\lambda_{post,i}$ were the number of new animal-to-human infections in study site *i* before and after LPM closure, t_b was the start date of LPM closure, and t_a and t_c were the start and end times of the time horizon for study site *i* in our analysis. We also assumed that the population in site *i* was subject to the a daily per capita force of infection, $\pi_{pre,i}/N_i$ and $\pi_{post,i}/N_i$, where N_i is the population in site *i*. We assessed the effect of LPM closure by the form $(1 - \lambda_{post,i}/\lambda_{pre,i}) \times 100\%$, which indicated the proportionate reduction in the number of infections after LPM closure.

For study sites with 2 LPM closure measures, we assumed that the new animal-to-human infections in these study sites followed a Poisson distribution with mean $\lambda_{1st,i} = p_i \cdot \pi_{1st,i}$ for the period before the first LPM closure, $\lambda_{2nd,i} = p_i \cdot \pi_{2nd,i}$ for the period during the first LPM

closure, and $\lambda_{3rd,i} = p_i \cdot \pi_{3rd,i}$ for the period after the first LPM closure (or during the second LPM closure). The transmission function was given by:

$$h_{A,i}(t) = \begin{cases} F(t) \cdot \lambda_{1st,i} & t \in [t_0, t_1) \\ F(t-t_1) \cdot \lambda_{2nd,i} + [1 - F(t-t_1)] \cdot \lambda_{1st,i} & t \in [t_1, t_2) \\ F(t-t_2) \cdot \lambda_{3rd,i} + [1 - F(t-t_2)] \cdot \left(\lambda_{1st,i} + \lambda_{2nd,i}\right) & t \in [t_2, t_3) \end{cases}$$

where $\lambda_{1st,i}$, $\lambda_{2nd,i}$, and $\lambda_{3rd,i}$ were the number of new animal-to-human infections in study site i; t_1 and t_2 were the start dates of the first and second LPM closure; and t_0 and t_3 were the start and end times for study site *i* in the analysis. The population in site *i* was assumed to be subject to a daily per capita force of infection $\pi_{1st,i}/N_i$, $\pi_{2nd,i}/N_i$ and $\pi_{3rd,i}/N_i$. $(1-\lambda_{2nd,i}/\lambda_{1st,i}) \times 100\%$ and $(1-\lambda_{3rd,i}/\lambda_{2nd,i}) \times 100\%$ were used to evaluate the effect of different period of LPMs closure.

Human-to-Human Transmission Model

In the human-to-human transmission model, we assumed that human infections had an infectiousness profile following a Poisson distribution with mean serial interval (S_p) of H7N9 infection. The human-to-human transmission model was defined as follows:

$$h_{H,i}(t) = \sum_{j=1}^{\min(k.t)} R_e N(t-j-1) \frac{S_p^j e^{-S_p}}{j!} \qquad t \in (0, t_n)$$

where *N(t)* was the number of new human infections each day chosen from a Poisson distribution with mean $h_{A,i}(t)$+$h_{H,i}(t)$. The expected number of cases on day *t* was given by:

$$h_i(t) = \begin{cases} h_{A,i}(t) & t = 0 \\ \sum_{j=1}^{\min(k.t)} R_e N(t-j-1) \frac{S_p^j e^{-S_p}}{j!} + h_{A,i}(t) & t \in (0, t_n) \end{cases}$$

where *k* is the maximum value the serial interval distribution can take; we fixed *k* = 14 days in main analyses.

We used a Markov chain Monte Carlo (MCMC) method to jointly estimate the expected number of new animal-to-human infections during each period and the effective reproduction number, on the basis of illness onset data. Each parameter was assumed to be positive and with noninformative uniform priors. We used a likelihood-based method to estimate epidemiologic parameters. The likelihood of a time series of observed human cases was:

$$L(\lambda_{pre,i},\lambda_{post,i},\mu,\sigma,R_e)=\prod_{t=0}^{t_n\text{-}1}\frac{h_i(t)^{N(t+1)}e^{-h_i(t)}}{N(t+1)!}$$

Sensitivity Analysis

Analysis of Influence of Proportion of Unreported Cases and Mean Serial Interval on the Effective Reproduction Number

We incorporated the proportion of unreported cases ($1–p_i$) and mean serial interval into the model in a sensitivity analysis. For patients with known exposure, an estimate of the serial interval is 7.5 days (95% CI 4.9–9.0) (*4*). We therefore assumed a serial interval of 7.5 days for our main analysis and tested a range of 5.5–9.5 days (*4*) during the sensitivity analysis; we adjusted for 4 days (*5*) for any potential delays such as symptom onset and case reports. Many unreported mild or asymptomatic H7N9 cases may have occurred (*6*), and this may potentially affect the pool of susceptible humans. Thus, the proportion of unreported cases was assumed to be 0.0, 0.2, 0.4, or 0.6, based on previous studies (*6*,*7*).

Analysis of Potential Effect of Absolute Humidity

The number of new animal-to-human infections and effective reproduction number on day *t* was extended to analyze how absolute humidity modulates the onset of H7N9 infection (*3*,*7*). Incorporating the potential effect of the seasonality of H7N9 epidemic into animal-to-human and human-to human transmission model, the extended models were

$$h_{A,i}(t)=\begin{cases}F(t)\cdot\alpha_{pre,i}\cdot Q(H_{t,i}) & t\in[t_a,t_b)\\ F(t)\cdot\alpha_{post,i}\cdot Q(H_{t,i})+(1-F(t))\cdot\alpha_{pre,i}\cdot Q(H_{t,i}) & t\in[t_b,t_c)\end{cases}$$

$$h_{H,i}(t)=\sum_{j=1}^{\min(k,t)}k\cdot(R_{0\min}+\exp(b+a\cdot H_{t,i}))\cdot \mathrm{N}(\mathrm{t-j+1})\cdot\frac{S_p^{\,j}e^{-S_p}}{j!}\qquad t\in(0,t_c)$$

where $H_{t,i}$ was the daily average absolute humidity on day *t* in study site *i*; $Q(H_{t,i})=1+exp(b+a\cdot H_{t,i})$ $(a>0)$ represented how absolute humidity modulated the force of infection (*8*,*9*); $\alpha_{pre,i}\cdot Q(H_{t,i})$ and $\alpha_{post,i}\cdot Q(H_{t,i})$ were the number of new animal-to-human infections on day *t* before and after LPM closure and followed a Poisson distribution; the ratio $1-\frac{\alpha_{pre,i}}{\alpha_{post,i}}$ (i.e., equivalent to $1-\lambda_{post,i}/\lambda_{pre,i}$ in the base model) represented the reduction in force of infection as a result of LPM closure; *k* was the susceptibility of population and was assumed to be approximately 100.0%; R_{0min} was the minimum basic reproduction number. For study sites with 2 LPM closure measures, the number of new animal-to-human infections on day *t* followed a Poisson distribution with mean $\alpha_{1st,i}\cdot Q(H_{t,i})$ for $\mathrm{t}\in[t_0,t_1)$, $\alpha_{2nd,i}\cdot Q(H_{t,i})$ for $\mathrm{t}\in[t_1,t_2)$, and $\alpha_{3rd,i}\cdot Q(H_{t,i})$ for $\mathrm{t}\in[t_2,t_3)$, where the ratio $1-\alpha_{2nd,i}/\alpha_{1st,i}$, and $1-\alpha_{3rd,i}/\alpha_{2nd,i}$ represented the reduction in force of infection resulting from LPM closure. Parameter *a* and *b* were assumed to be followed a semi-informative distribution (normal distribution with mean 0 and deviation 5).

References

1. Kucharski A, Mills H, Pinsent A, Fraser C, Van Kerkhove M, Donnelly CA, et al. Distinguishing between reservoir exposure and human-to-human transmission for emerging pathogens using case onset data. PLoS Curr. 2014;6:6. PubMed https://doi.org/10.1371/currents.outbreaks.e1473d9bfc99d080ca242139a06c455f

2. Kucharski AJ, Mills HL, Donnelly CA, Riley S. Transmission potential of influenza A(H7N9) virus, China, 2013–2014. Emerg Infect Dis. 2015;21:852–5. PubMed https://doi.org/10.3201/eid2105.141137

3. Yu H, Cowling BJ, Liao Q, Fang VF, Zhou S, Wu P, et al. Effect of closure of live poultry markets on poultry-to-person transmission of avian influenza A H7N9 virus: an ecological study. Lancet 2014;383:541–8. https://doi.org/10.1016/S0140-6736(13)61904-2

4. Yang Y, Zhang Y, Fang L, Halloran ME, Ma M, Liang S, et al. Household transmissibility of avian influenza A (H7N9) virus, China, February to May 2013 and October 2013 to March 2014. Euro Surveill. 2015;20:21056. PubMed https://doi.org/10.2807/1560-7917.ES2015.20.10.21056

5. Sun J, Gong Z, Lv H, Chen Z, Chai C, Liu S, et al. Comparison of characteristics between patients with H7N9 living in rural and urban areas of Zhejiang Province, China: a preliminary report. PLoS One. 2014;9:e93775. PubMed https://doi.org/10.1371/journal.pone.0093775

6. Xiang N, Bai T, Kang K, Yuan H, Zhou S, Ren R, et al. Sero-epidemiologic study of influenza A(H7N9) infection among exposed populations, China 2013-2014. Influenza Other Respir Viruses. 2017;11:170–6. PubMed https://doi.org/10.1111/irv.12435

7. Virlogeux V, Feng L, Tsang TK, Jiang H, Fang VJ, Qin Y, et al. Evaluation of animal-to-human and human-to-human transmission of influenza A (H7N9) virus in China, 2013-15. Sci Rep. 2018;8:552. PubMed https://doi.org/10.1038/s41598-017-17335-9

8. Shaman J, Kohn M. Absolute humidity modulates influenza survival, transmission, and seasonality. Proc Natl Acad Sci U S A. 2009;106:3243–8. PubMed https://doi.org/10.1073/pnas.0806852106

9. Shaman J, Pitzer VE, Viboud C, Grenfell BT, Lipsitch M. Absolute humidity and the seasonal onset of influenza in the continental United States. PLoS Biol. 2010;8:e1000316. PubMed https://doi.org/10.1371/journal.pbio.1000316

Appendix Table 1. Action lists for live poultry market closures in China, 2013–2017

Action Code	Action	Frequency of LPM
1	Close 1 d per month	Recursive
2	Close 1 d per month, disinfection on close day	Recursive
3	Close 1 d per month, clean 1 d per week	Recursive
4	Close 1 d per month, disinfection per week	Recursive
5	Close 1 d per month, clean per day, disinfection per week	Recursive
6	Close 1 d per month, clean 1 d per week, No stay overnight	Recursive
7	Close 2 d per month	Recursive
8	Close 2 d per month, clean 1 d per week	Recursive
9	Close 1 d per 2 weeks, disinfection on the close day	Recursive
10	Close in 3 fixed time	Recursive
11	Close 3 d per month	Recursive
12	Close 3 d per month, clean 1 d per week	Recursive
13	Close 1 d per week	Recursive
14	Close 1 d per week in wholesale market, close 1 d per 2 weeks in retail level	Recursive
15	Close 1 d per week, disinfection on close day	Recursive
16	Close 2 d per week	Recursive
17	Close 3 d per month, disinfection per day	Recursive
18	Disinfection per 2 weeks	Recursive
19	Disinfection per week	Recursive
20	Disinfection per week, clean per day	Recursive
21	Disinfection per day	Recursive
22	Disinfection and clean per day	Recursive
23	Disinfection twice per day	Recursive
24	Close 1 d to clean and disinfect	Short
25	Close 3 d	Short
26	Close 5 d	Short
27	Close 1 week	Short
28	Close 2 weeks	Short
29	Temporarily close in >1 markets	Long
30	Temporarily close	Long
31	Gradually cancel live poultry trading	Long
32	Permanent close	Permanent

Appendix Table 2. Regression coefficients of fixed effect of various types and closing levels of live poultry market closure on daily incidence rate using general linear mixed model across waves, China

Types and closing levels of	Wave	Category	β_i	Standard error	Z value	p value
Proportion of closing days	1	No closure	1.376	0.362	3.797	<0.001
		<25% closing days	–1.653	0.722	–2.287	0.022
	2	No closure	0.244	0.251	0.973	0.331
		25%–75% closing days	–4.479	1.010	–4.434	<0.001
		>75% closing days	–1.350	0.248	–5.446	<0.001
	3	No closure	0.698	0.226	3.094	0.002
		<25% closing days	–3.043	0.717	–4.244	<0.001
		25%–75% closing days	–3.204	1.022	–3.135	0.002
		>75% closing days	–1.972	0.380	–5.183	<0.001
	4	No closure	1.489	0.368	4.049	0.175
		>75% closing days	–0.613	0.452	–1.355	<0.001
	5	No closure	0.526	0.131	4.006	<0.001
		<25% closing days	–1.652	0.240	–4.676	<0.001
		25%–75% closing days	–1.361	0.209	–6.514	<0.001
		>75% closing days	–1.131	0.182	–6.232	<0.001
Type of closure	1	No closure	4.499	0.727	6.191	<0.001
		Before closure	1.610	0.784	2.052	0.040
		Permanent closure	0.847	0.784	1.081	0.280
	2	No closure	1.904	0.376	5.059	<0.001
		Before closure	0.955	0.369	2.585	0.010
		Long-period closure	–0.876	0.371	–2.361	0.018
		Permanent closure	–2.292	0.703	–3.259	0.001
	3	No closure	3.121	0.395	7.911	<0.001
		Before closure	1.149	0.364	3.158	0.002
		Long-period closure	–3.019	1.062	–2.842	0.005
		Permanent closure	–0.859	1.495	–1.735	0.083
	4	No closure	4.499	0.727	6.191	<0.001
		Before closure	1.610	0.784	2.052	0.040
		Permanent closure	0.847	0.784	1.081	0.280
	5	No closure	2.064	0.201	10.289	<0.001
		Before closure	0.491	0.177	2.777	0.006
		Short-period closure	–0.829	0.321	–2.586	0.010
		Long-period closure	–0.744	0.226	–3.289	0.001
		Permanent closure	–0.729	0.266	–2.737	0.006

Appendix Table 3. Multiple comparisons of the reduction in number of infections before and after live poultry market closure among study sites with different types of live poultry market closure in the 2016–17 H7N9 epidemic wave, China.

Type of live poultry market closure		Mean difference of the reduction in number of	p value
Permanent closure	Long-period closure	45.0 (40.3–49.7)	<0.001
	Short-period closure	28.2 (23.8–32.7)	<0.001
	Recursive closure	15.3 (11.3–19.3)	<0.001
Long-period closure	Short-period closure	12.9 (10.4–15.4)	<0.001
	Recursive closure	29.7 (26.8–32.7)	<0.001
Short-period closure	Recursive closure	16.8 (13.3–20.3)	<0.001

*Difference in the effectiveness of the 2 LPM interventions presented in the first and second columns.

Appendix Table 4. Estimates of the effective reproduction number in the 2016–17 epidemic wave of H7N9, China, considering the impact of the proportion of unreported cases and the mean serial interval on the effective reproduction number.

Parameters		Reproduction number (95% CI)
Proportion of unreported cases	0.0	0.147 (0.034–0.285)
	0.2	0.151 (0.042–0.283)
	0.4	0.125 (0.033–0.232)
	0.6	0.162 (0.063–0.265)
Mean serial interval (S_p)	5.5 d	0.156 (0.043–0.298)
	6.5 d	0.150 (0.037–0.292)
	7.5 d	0.147 (0.034–0.285)
	8.5 d	0.138 (0.029–0.273)
	9.5 d	0.131 (0.021–0.274)

Appendix Table 5. Comparison of parameter estimates from models with and without an effect of absolute humidity in the 2016–17 H7N9 epidemic wave, China.

Site	Type of closures	Reduction in force of infection due to live poultry market closure			
		Model without an effect of absolute		Model with an effect of	
Site 1	Permanent	97.0 (94.0–100.0)		97.0 (93.0–100.0)	
	Site 2	Long	90.0 (87.0–95.0)		90.0
	Site 3	Long	92.0 (87.0–100.0)		90.0
	Site 4	Long	95.0 (90.0–100.0)		94.0
	Site 5	Long	98.0 (96.0–100.0)		98.0
	Site 6	Long	48.0 (35.0–81.0)		50.0
	Site 7	Recursive	34.0 (15.0–70.0)		36.0
		Short	73.0 (53.0–77.0)		70.0
	Site 8	Short	96.0 (93.0–99.0)		96.0
	Site 9	Long	84.0 (75.0–96.0)		84.0
	Site 10	Long	89.0 (80.0–99.0)		90.0
	Site 11	Long	92.0 (84.0–99.0)		92.0
	Site 12	Long	78.0 (72.0–92.0)		78.0
	Site 13	Long	86.0 (83.0–87.0)		86.0
	Site 14	Long	92.0 (86.0–100.0)		93.0
	Site 15	Short	95.0 (92.0–98.0)		95.0
	Site 16	Recursive	71.0 (47.0–97.0)		73.0
Site 17	Long	84.0 (79.0–94.0)		84.0 (78.0–94.0)	
Minimal basic reproduction number (95% CrI)					
Parameter*				0.061 (0.004–0.147)	
	a			0	2.89
b			∞	–0.85 (–6.52–+4.76)	

*a and b are key parameters used to modulate the impact of absolute humidity on the force of infection.

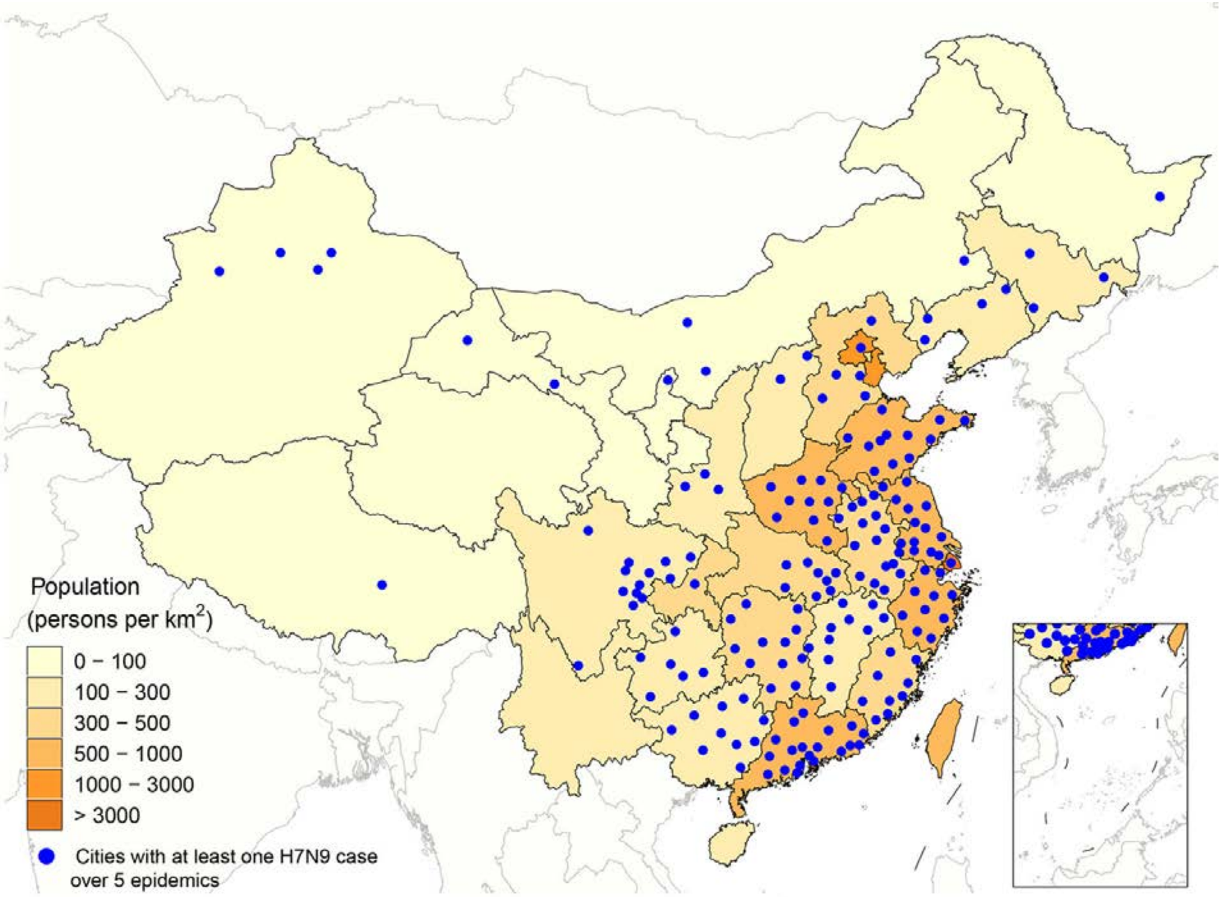

Appendix Figure 1. Geographical distribution of predefined locations when using search engines to update the live poultry market closure measures, China. The blue dots refer to the cities with ≥1 H7N9 case over 5 epidemic waves.

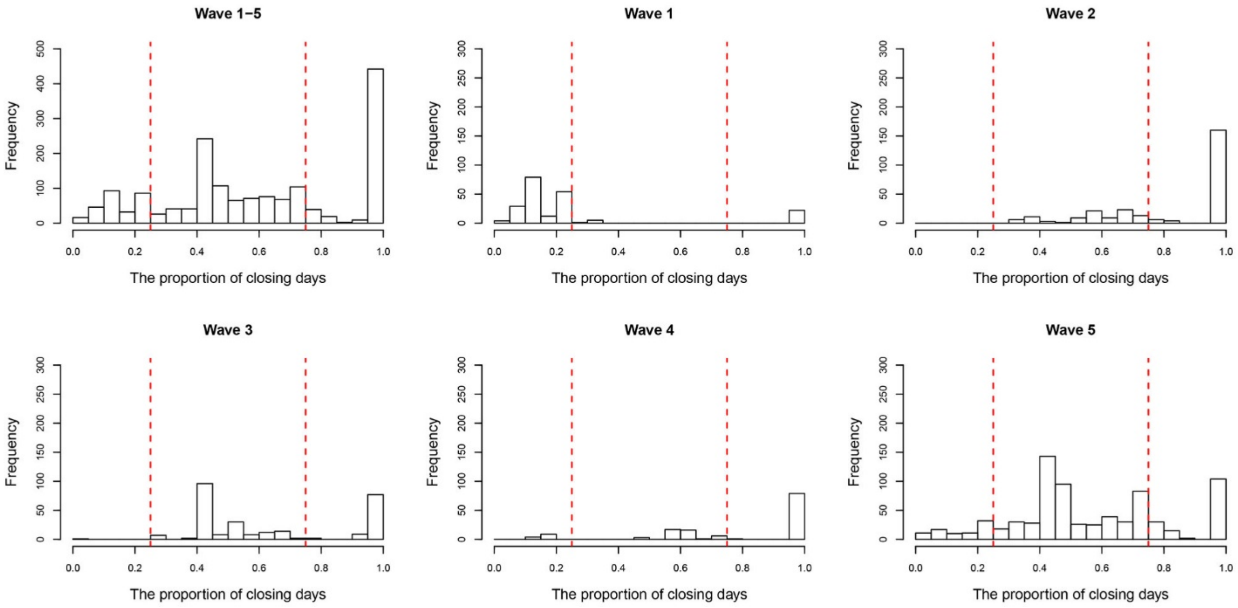

Appendix Figure 2. Distribution of the proportion of days of closing out of the total epidemic wave duration in 2013–2017. The red vertical lines refer to 25% and 75% of the total epidemic wave duration.

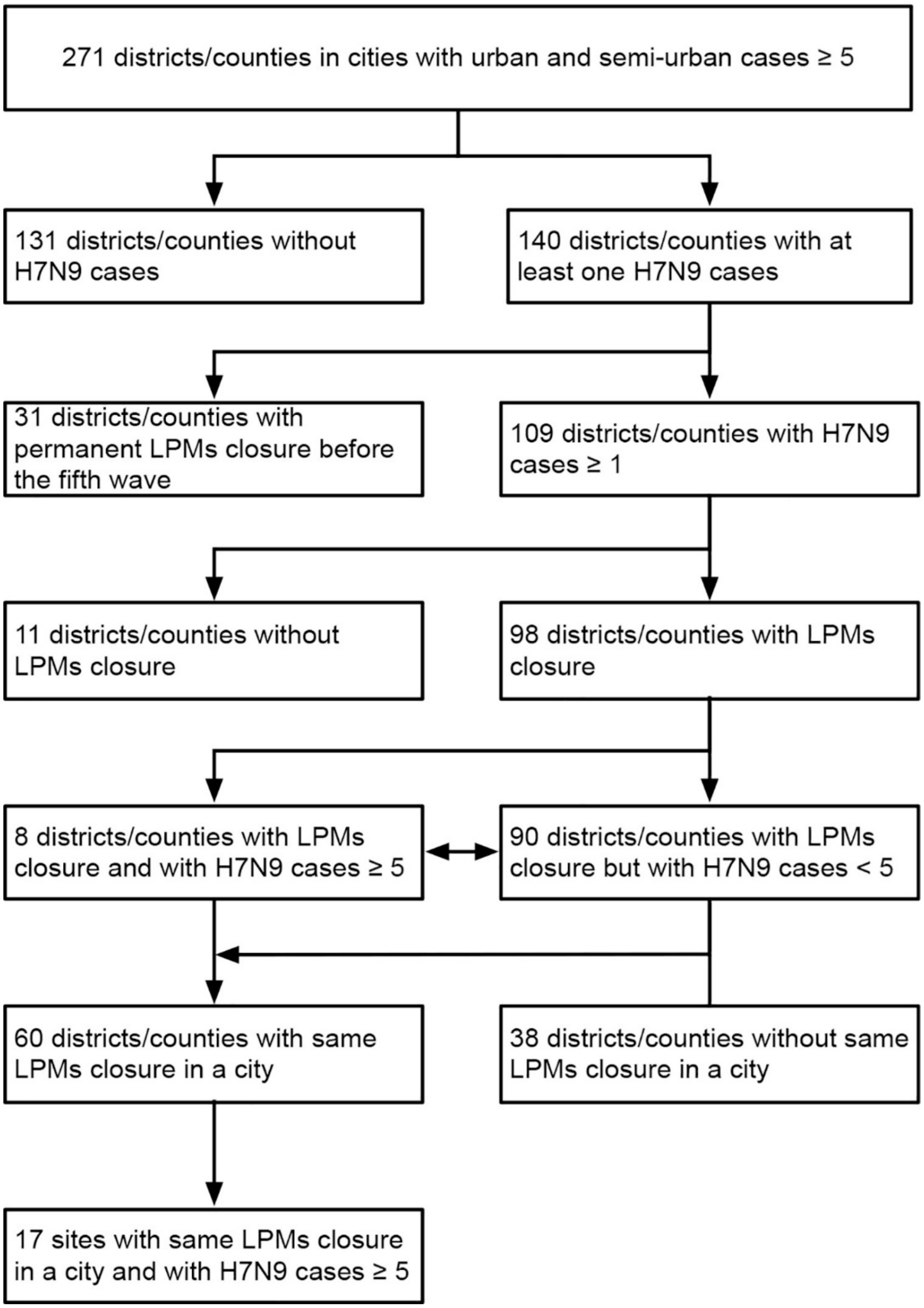

Appendix Figure 3. Flowchart of the selection of sites in quantitative evaluation of live poultry markets closure in the 2016–17 H7N9 epidemic wave, China.

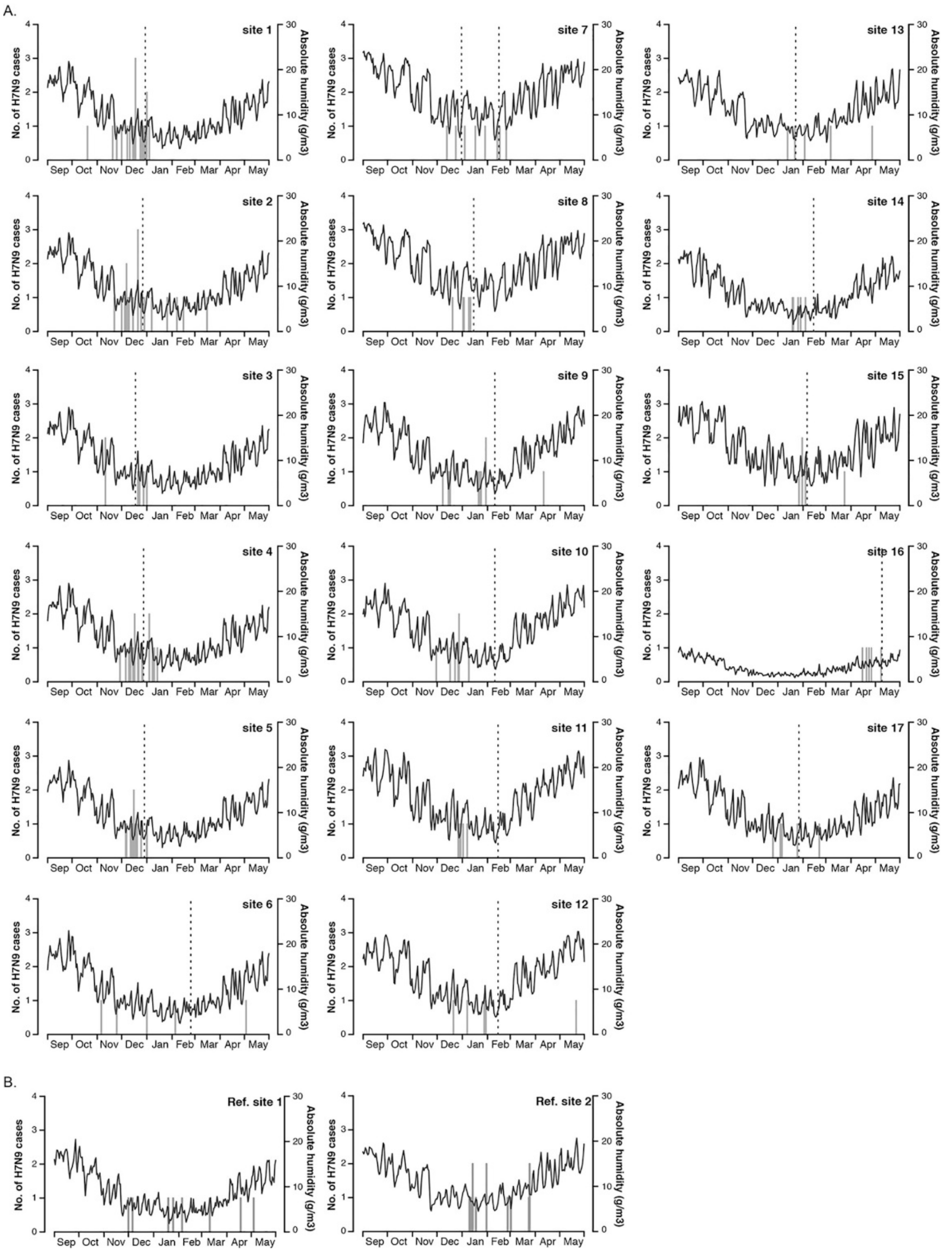

Appendix Figure 4. Time series of absolute humidity and illness onset of human H7N9 cases in sites with and without live poultry market closures, China. The gray bars indicate the number of cases with onsets on that day. Red vertical lines indicate the start date of live poultry market closures in each study site. The blue curves refer to the daily average absolute humidity in each site. A) Absolute humidity and illness onset of human cases in 17 study sites with closures and B) in 2 reference sites without closures.

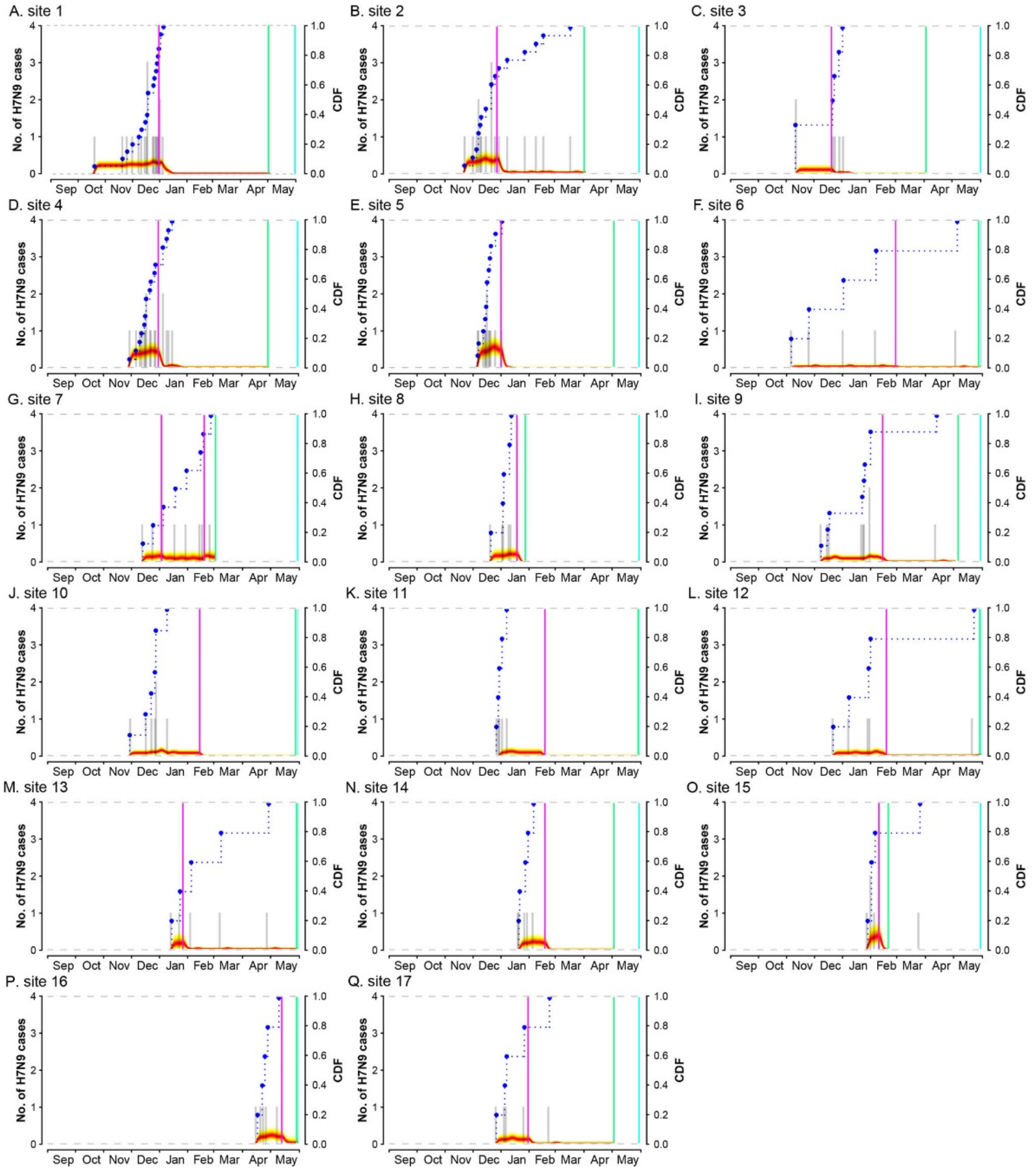

Appendix Figure 5. Dates of H7N9 cases and posterior estimates of the expected daily number of illness onsets of cases in 17 sites in the 2016–17 H7N9 epidemic wave. The gray bars indicate the number of cases with onsets on that day. The magenta vertical lines refer to the start date of live poultry market closures, the green vertical lines indicate the end date of live poultry market closures, and the cyan vertical lines indicate the last date used in analyses. The blue points and dashed lines represent the cumulative distribution function (CDF) of H7N9 epidemics in humans. The red and yellow colors in each panel refer to whether the value of posterior estimates is included within the 95th prediction intervals on a given day.

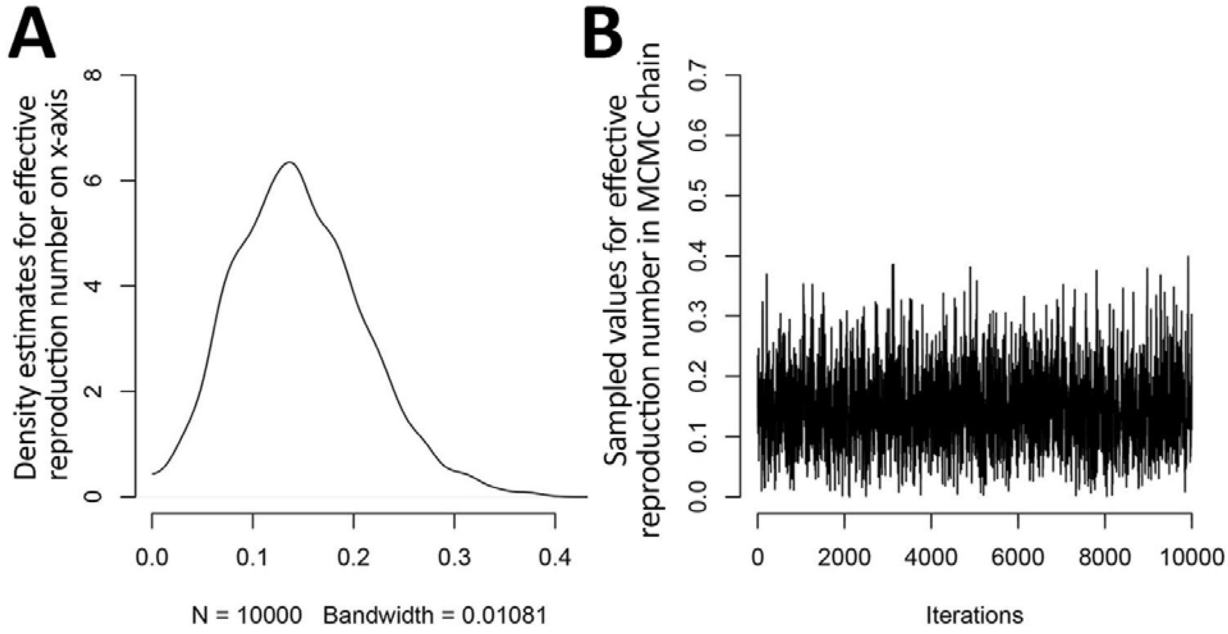

Appendix Figure 6. The posterior distribution and Markov chain Monte Carlo (MCMC) sampling process of effective reproduction number. A) The posterior distribution for effective reproduction number. B) The MCMC sampling process of effective reproduction number.

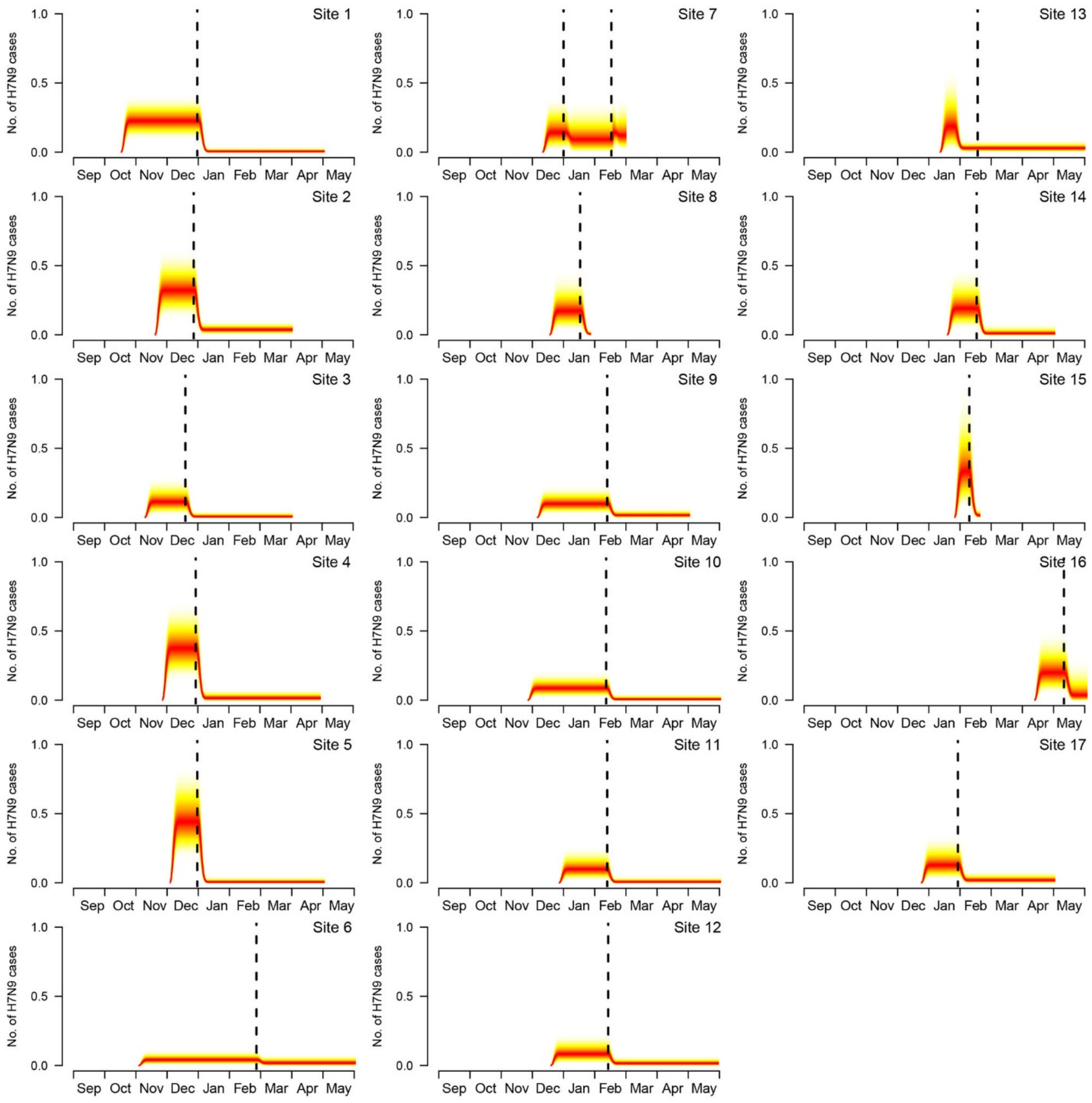

Appendix Figure 7. Posterior estimates of the expected daily number of illness onsets of H7N9 cases resulting from animal-to-human transmission in 17 sites in the 2016-17 epidemic wave, China. The red and yellow colors in each panel refer to whether the value of these estimates is included within the 95th prediction intervals on a given day. Black vertical lines indicate the start date of live poultry market closures in each study site.

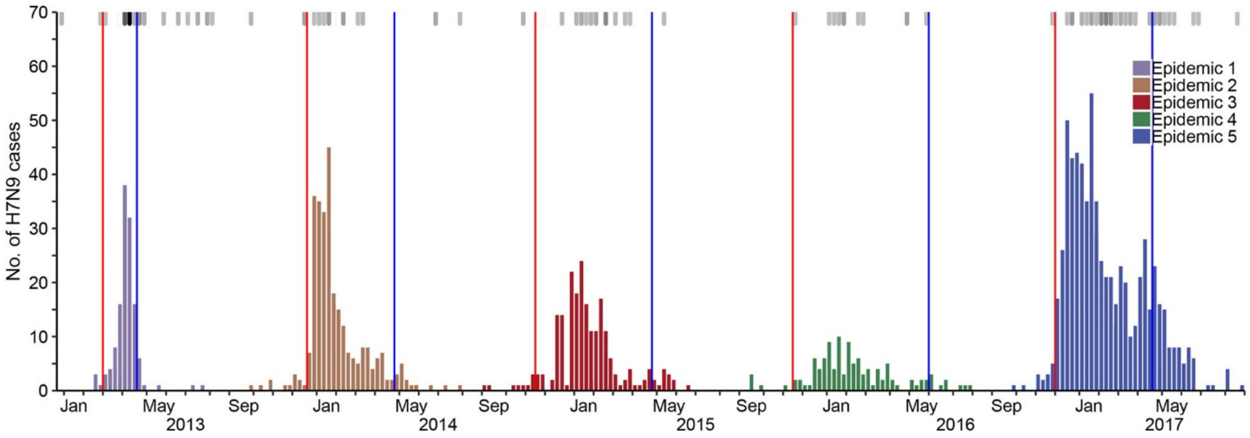

Appendix Figure 8. Temporal pattern of laboratory-confirmed H7N9 cases and the implementation of live poultry market interventions in mainland China across epidemic waves. The small and partially transparent lines represent the implementation of live poultry market measures; darker colors indicate higher frequency of the implementation of live poultry market interventions. Red vertical lines and blue vertical lines refer to the 5th and 95th percentiles of the days of onset of illness in each epidemic wave. The epidemic peak of H7N9 in each wave appeared to coincide with the period in which the closure measures were implemented.
